# Successful removal of an accidentally swallowed press‐through package sheet using a detachable snare: A case report

**DOI:** 10.1002/deo2.41

**Published:** 2021-09-07

**Authors:** Fumihiro Mawatari, Naohiro Komatsu, Ryosaku Oshiro, Tetsuhiko Arima, Sachiko Fukuda, Yoshiko Kita, Aiko Fukahori, Kazuhiko Nakao

**Affiliations:** ^1^ Department of Gastroenterology Juzenkai Hospital Nagasaki Japan; ^2^ Department of Gastroenterology and Hepatology Graduate School of Biomedical Sciences Nagasaki University Nagasaki Japan; ^3^ Department of Internal Medicine Juzenkai Hospital Nagasaki Japan

**Keywords:** detachable snare, endoscopic removal, foreign body, grasping forceps, press‐through package sheet

## Abstract

Accidental swallowing of press‐through package (PTP) sheets that could cause esophageal perforation is commonly encountered in emergency departments requiring early detection and removal. We report a case in which an accidentally swallowed PTP sheet was removed from the esophagus using a detachable snare after usual endoscopic methods proved ineffective. A Japanese woman in her 60s visited the emergency department with a persistent sore throat. Cervicothoracic computed tomography revealed presence of a PTP sheet in the cervical esophagus, and emergency endoscopy was performed. Pre‐endoscopy simulations using a sheet identical to the one swallowed by the patient indicated that the sheet would not have been retrievable using an overtube (its inner diameter was smaller than the sheet's diameter) and grasping forceps (they slipped off the sheet). It was successfully removed using a detachable snare, a device normally employed in colorectal polypectomy, allowing us to ligate the end of the sheet and pull it into the overtube. To the best of our knowledge, this is the first report of endoscopic removal of a PTP sheet from the esophagus using a detachable snare. We suggest that this novel method would facilitate removal of esophageal PTP sheets.

## INTRODUCTION

Accidentally swallowed foreign objects are common causes of emergency department visits.^1^ In Japan, press‐through package (PTP) sheets are the most frequently swallowed foreign objects encountered in emergency departments.^2^ PTP sheets are usually retrieved from the esophagus by pulling them into a transparent hood or an overtube with forceps.^2^ However, we have experienced cases where PTP sheets were too large to fit into these devices, making treatment difficult. Herein, we report a case in which a detachable snare for colorectal polypectomy was used to successfully and safely remove a large PTP sheet from the esophagus.

## CASE REPORT

A Japanese woman in her 60s complained of sore throat at home following intake of her regular medications for several common diseases after dinner. She independently performed her activities of daily living. Owing to persistent symptoms, she was referred to our emergency department. On examination, her blood pressure was 162/80 mm Hg, and her pulse rate was 67 beats/min. There was no evidence of cerebrovascular disease or dementia. Neck swelling or subcutaneous emphysema is not suspected. No other specific findings were found.

**Figure 1:**
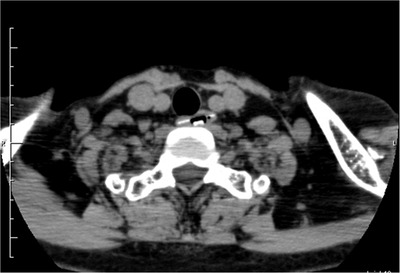
Cervicothoracic computed tomography reveals a 17 mm^2^ PTP sheet inside the cervical esophagus

**Figure 2:**
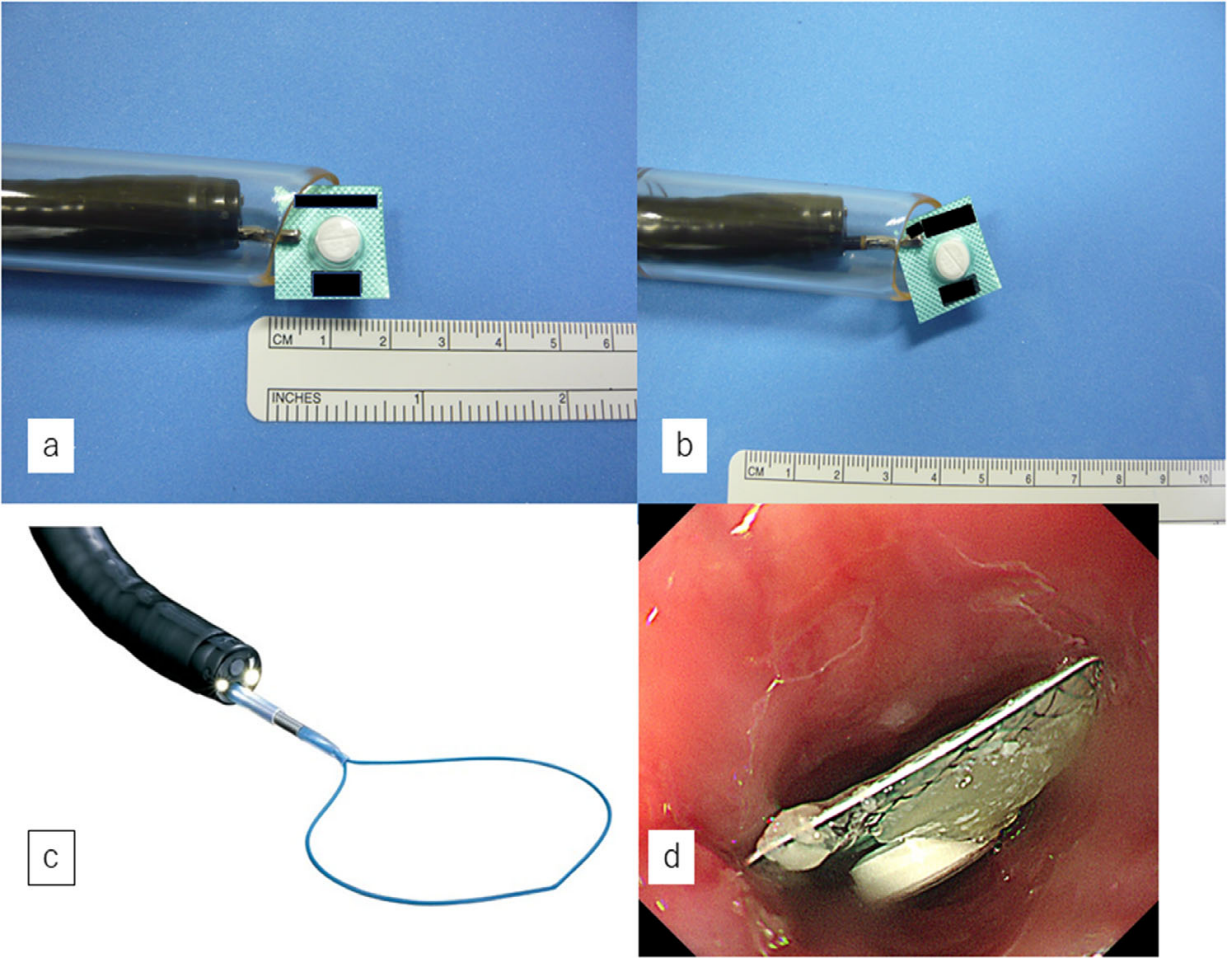
Simulations before endoscopy. (**a)** The diameter of the press‐through package sheet is larger than the inner diameter of the overtube. (**b)** Attempting to pull the sheet into the overtube after grabbing it diagonally with forceps is not successful. (**c)** The detachable snare used for endoscopy in this study. (**d)** Esophagoscopy showing a press‐through package sheet (17 × 17 mm) in the upper esophagus

On arrival, cervicothoracic computed tomography revealed a 17 mm^2^ PTP sheet inside the cervical esophagus. (Figure 1) There was no obvious perforation, and urgent endoscopy was performed on the same day. Before endoscopy, a PTP sheet identical to the one swallowed was obtained from a family member for simulations using an overtube (MD‐48518: Sumitomo Bakelite, Tokyo, Japan) and grasping forceps (FG‐42 L‐1: Olympus Medical Systems Co., Tokyo, Japan). The inner diameter of the overtube (15 mm) was smaller than that of the sheet. (Figure 2a). Attempting to grab the sheet diagonally with grasping forceps and pull it into the overtube was also unsuccessful: the forceps slipped off the sheet's hard surface (Figure 2b).

**Figure 3:**
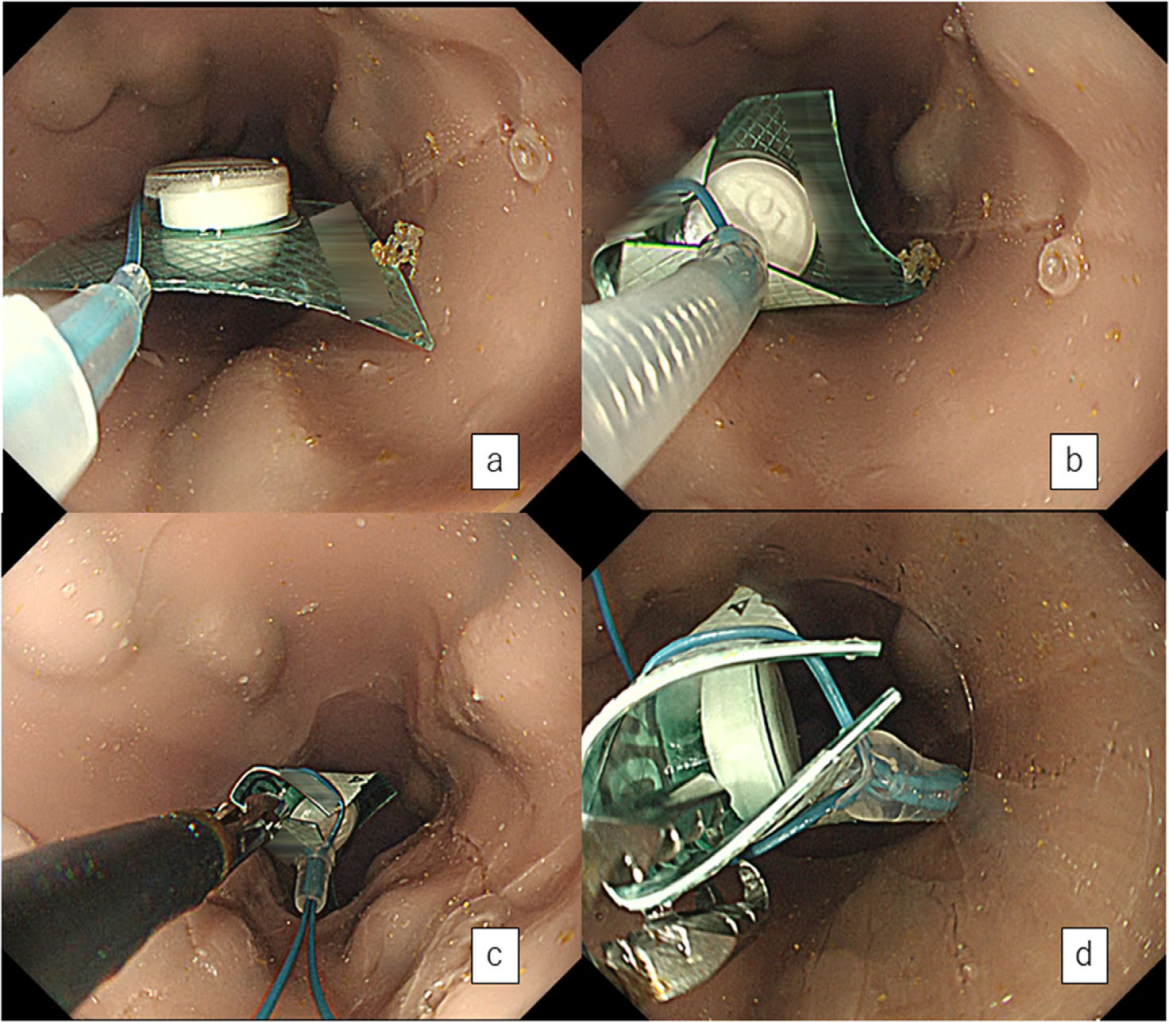
Procedure for removal of the press‐through package (PTP) sheet from the esophagus using an indwelling snare. (**a and b)** The detachable snare is opened, with the opening parallel to the esophagus, and the left side of the PTP sheet is ligated. (**c)** The ligated side of the PTP sheet is grasped with grasping forceps. (**d)** The sheet was pulled into the overtube for removal

Therefore, we considered retrieving the PTP sheet using a detachable snare (HX‐20Q‐1, Olympus Medical Systems Co., Tokyo, Japan) (Figure 2c). We confirmed its viability for ligating one side of the sheet with a detachable snare. The PTP sheet was deemed retrievable. Upper endoscopy was performed under midazolam sedation, confirming the sheet's presence in the cervical esophagus (Figure 2d). Since the sheet was just below the esophageal inlet, an overtube almost could not have been inserted. Therefore, air could not be pumped into the esophagus, and the sheet did not move. We could not obtain clean endoscopic images. Thus, we used models to illustrate what was done (Figures 3a‐3d, video 1). First, the detachable snare was opened, with its opening parallel to the esophagus, and the sheet's left side was ligated, paying attention not to damage the mucosa. (Figures 3a and 3b). Although the detachable snare was flexible, ligation was relatively easy owing to its side‐by‐side alignment with the esophageal opening. Second, the ligated side of the sheet was grasped with forceps (Figure 3c) and pulled into an overtube (Figure 3d). Afterwards, the endoscope was reinserted, and the esophagus was observed. A small amount of bleeding was noted from the mucosa where the PTP was fixed. However, there was no obvious mucosal laceration.

**Video 1:** 

**Figure 4:**
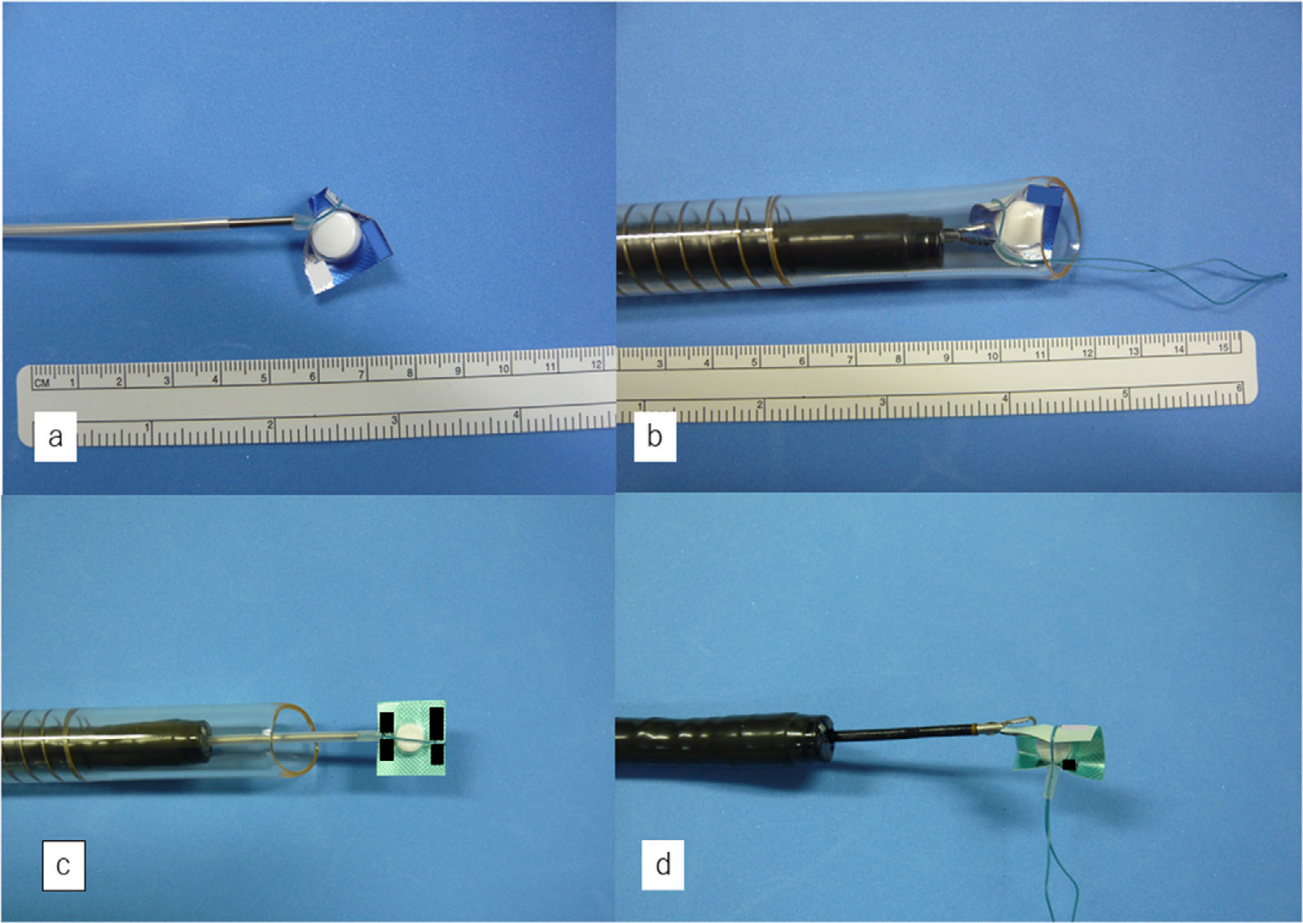
Removal of a different type of press‐through package (PTP) sheet and use of a different ligation site. (**a and b)** A detachable snare is used to remove a hard, large PTP sheet with a large tablet. (**c and d)** By changing the ligation site, all edges of the sheet can be folded inward, and it might be possible to remove the PTP sheet without the use of an overtube or large diameter tip hood

Furthermore, we determined whether our detachable snare technique was applicable to other PTP sheet types. Among six cases of accidental swallowing treated at our hospital in the past 5 years, five PTP sheet types were examined. These five types had different sheet sizes, tablet sizes, and surface coatings. We used an upper endoscope, overtubes, detachable snares, and grasping forceps to perform the experiment. Four of the five sheets could be ligated using detachable snares, easily pulled into overtubes, and removed with grasping forceps. Even in difficult‐to‐treat cases involving large, hard sheets with large tablets, our technique was successful (Figures 4a and 4b). However, in only one case, the sheet's surface was coated with a smooth vinyl, and our snare ligation technique was unsuccessful after several attempts.

Lastly, using a PTP sheet identical to the one in this case, we examined whether modification of the detachable snare technique could obviate the need for an overtube or tip hood. If the PTP sheet could be ligated at its center and deformed as shown in the photograph, this may be possible (Figures 4c and 4d).

This case report was approved by the Ethics Committee of Juzenkai Hospital (J2020‐10). The patient provided written consent for the procedure and this report's publication.

## DISCUSSION

This report describes a case of accidental swallowing of a large, hard PTP sheet that subsequently lodged in the cervical esophagus. Since retrieval into an overtube was difficult, we ligated the end of the sheet using a detachable snare. We then grasped the ligated side of the sheet with grasping forceps and pulled the sheet into an overtube for removal. The PTP edge was folded inward, allowing the sheet to be pulled smoothly into an overtube (Figures 3a‐3d, video 1). At first, we considered using a polypectomy snare to suture and pull the PTP sheet into an overtube. However, the contralateral edge of the sheet would have damaged the mucosa as we pulled it in (supporting information 2).

PTP sheets are usually removed from the esophagus by retrieval into transparent tip hoods or overtubes.^2^, ^3^, ^4^ However, this might be challenging, especially when the sheet is larger than the inner diameter of the tip hood or overtube or rigid, similar to this case. We encountered these problems in two of the six cases treated at our hospital within the last 5 years. In another case, we unsuccessfully attempted to extract the sheet using retrieval net, and it was eventually recovered by other means. Special tip hoods, such as endoscopic skirts, have been used in difficult‐to‐treat cases, with good results.^5^ However, such tools are often not readily available. Cases of accidental PTP sheet ingestion should be managed using instruments regularly available in endoscopy rooms.

The detachable snare used in this study was developed by Hachisu in 1989 for the treatment of post‐polypectomy hemorrhage.^6^ Most endoscopy facilities in Japan have detachable snares, and their staff are familiar with their uses. Detachable snares have been used to compress migrated esophageal self‐expandable metal stents for endoscopic removal^7^, ^8^ and to fold and compress a large, flat, sharp‐edged fish bone in the esophagus, allowing its safe removal.^9^ In the present case, we used a detachable snare to ligate the PTP sheet that was accidentally swallowed. To the best of our knowledge, this is the first report in which this device was used for this purpose. Care should be taken not to pinch the mucosa when stitching with a snare.

Using detachable snares for removal of PTP sheets from the esophagus is presently unapproved and thus should be carefully considered. However, this method may be considered in difficult‐to‐treat cases. The patient and family should be informed in advance about using a detachable snare, and pretreatment simulations should be performed to ensure that the type of sheet swallowed could be ligated by the snare.

Currently, a large‐caliber, soft oblique cap has been proposed as the most reliable and safe method for removing a PTP sheet from the esophagus.^1^ We suggest that our detachable snare technique would become the future method of choice for removing PTP sheets from the esophagus. Using this method, PTP sheets removal by only using snare may be adopted in children and patients with spinal and otolaryngological diseases who cannot endure large‐diameter hoods or overtubes. Since the results of only one case are presented here, further cases are required. In addition, further refinement of the method to establish it as a first‐line treatment might be needed.

## CONFLICT OF INTEREST

The authors have no conflict of interest to declare.

## FUNDING INFORMATION

None.

## Supporting information


**Figure S1**. Actual endoscopic view at the time of PTP removal.

**Figure S2**. Endoscopic PTP extraction. The difference between using a polypectomy snare and detachable snare.

## References

[1] Sugawa C . Endoscopic management of foreign bodies in the upper gastrointestinal tract: A review. World J Gastrointest Endosc 2014; 6: 475–81. 25324918 10.4253/wjge.v6.i10.475 PMC4198392

[2] Kamiya KJL , Hosoe N , Takabayashi K , *et al*. Endoscopic removal of foreign bodies: A retrospective study in Japan. World J Gastrointest Endosc 2020; 12: 33. 31942232 10.4253/wjge.v12.i1.33 PMC6939123

[3] Seo YS. Removal of press‐through‐packs impacted in the upper esophagus using an overtube. World J Gastroenterol 2006; 12: 5909–12. 17007065 10.3748/wjg.v12.i36.5909 PMC4100680

[4] Chávez Rossell M . New technique for safe removal of impacted foreign bodies in the upper gastrointestinal tract using reusable variceal “cap” (cup, cap or cylinder). Rev Gastroenterol Peru 2012; 32: 150–6. 23023177

[5] Takenaka Y , Kakemura T , Sato K , *et al*. A case of safe extraction of press‐through‐package using a hooded skirt mounted to the tip of an endoscope. Prog Dig Endosc 2011; 78: 78–9.

[6] Hachisu T . A new detachable snare for hemostasis in the removal of large polyps or other elevated lesions. Surg Endosc 1991; 5: 70–74. 1948617 10.1007/BF00316840

[7] An H‐J , Lee H‐Y , Kim B‐W , *et al*. Endoscopic removal of a migrated esophageal self‐expandable metal stent after compression with detachable snares through an intact esophageal stent. Gastrointest Endosc 2010; 71: 205–7. 19640522 10.1016/j.gie.2009.06.008

[8] Yip HC , Chiu PW , Ng EK . Endoscopic retrieval of a distally migrated stent using detachable snares. Endoscopy 2013; 45: E420–1. 24338170 10.1055/s-0030-1256586

[9] Lee JS , Chun HJ , Lee JM , *et al*. Salvage technique for endoscopic removal of a sharp fish bone impacted in the esophagus using a transparent cap and detachable snares. Korean J Gastroenterol 2013; 61: 215–8. 23624736 10.4166/kjg.2013.61.4.215

