# Supplementary figures and images for: Successful removal of an accidentally swallowed press‐through package sheet using a detachable snare: A case report
Source: DEN Open. 2021 Sep 7;2(1):e41. doi: 10.1002/deo2.41 (PMC8828184; doi:10.1002/deo2.41)

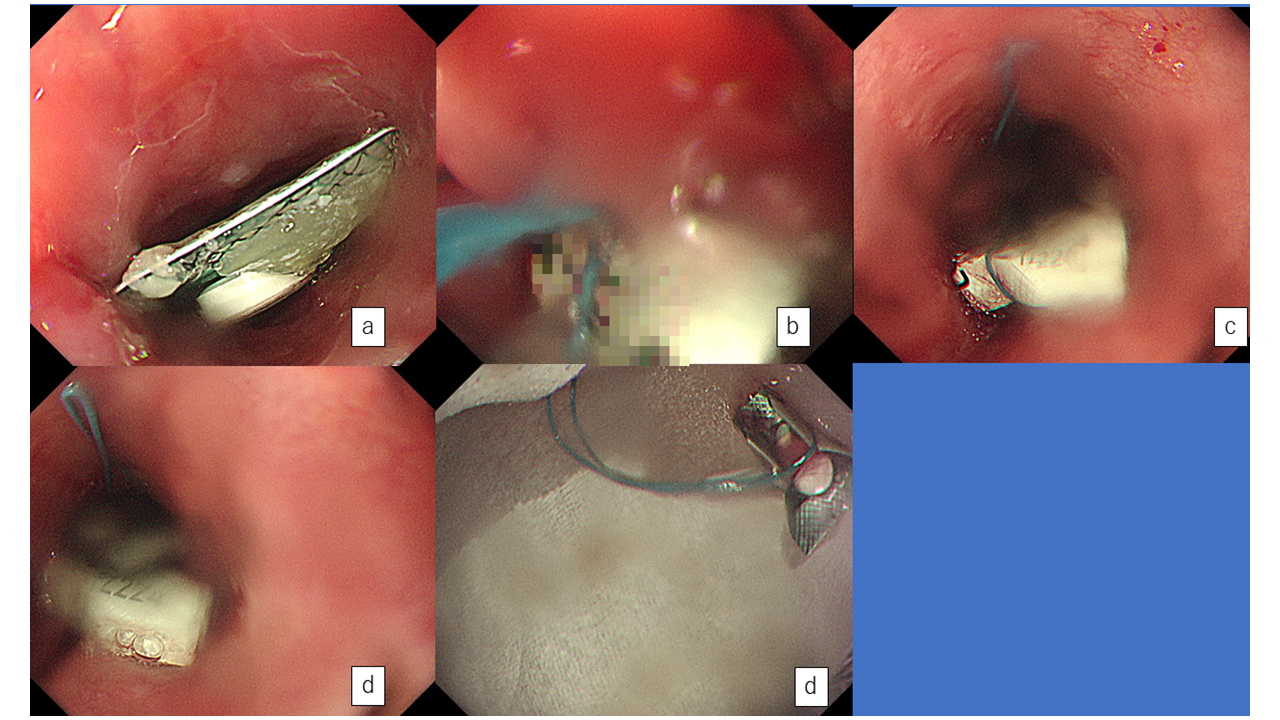

Supplement: Supplementary file 1 — Figure S1. Actual endoscopic view at the time of PTP removal. [file DEO2-2-e41-s002.TIF]

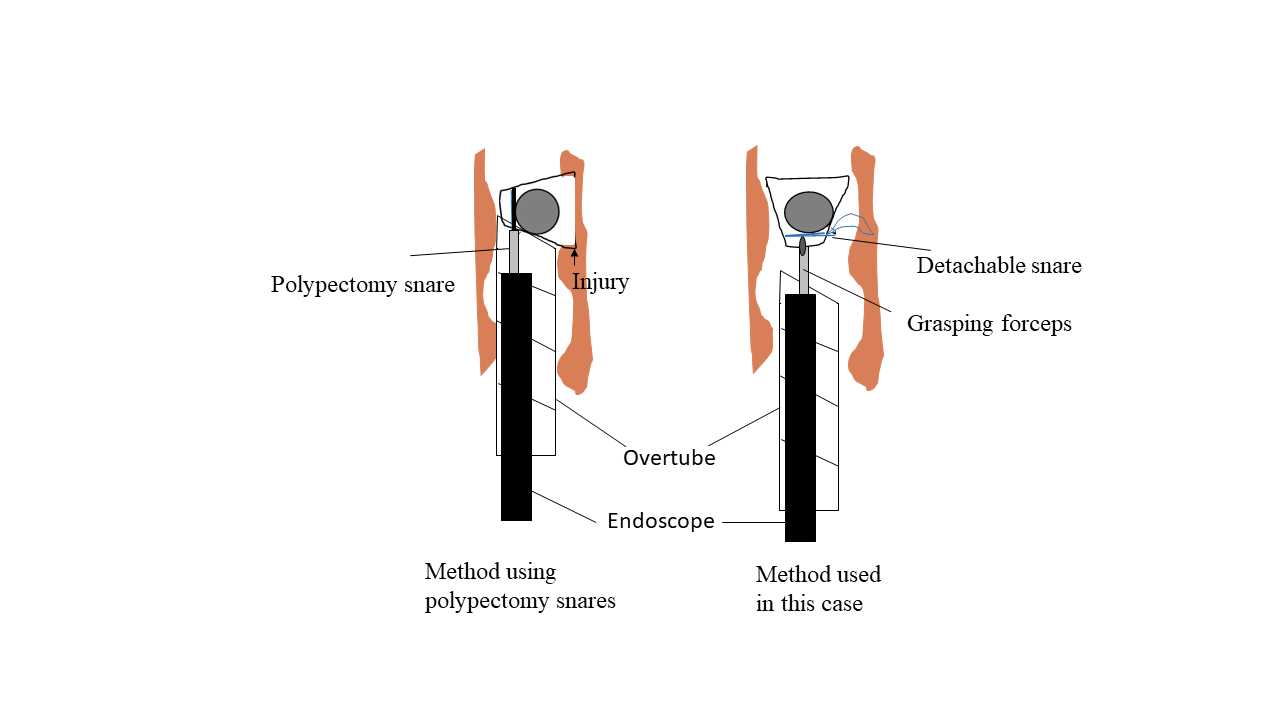

Supplement: Supplementary file 2 — Figure S2. Endoscopic PTP extraction. The difference between using a polypectomy snare and detachable snare. [file DEO2-2-e41-s001.tif]
